# A longitudinal qualitative exploration of Victorian healthcare workers’ and organisations’ evolving views and experiences during COVID-19

**DOI:** 10.1186/s12913-024-11067-z

**Published:** 2024-05-07

**Authors:** Sarah L. McGuinness, Owen Eades, Shannon Zhong, Sharon Clifford, Jane Fisher, Helen L. Kelsall, Maggie Kirkman, Grant Russell, Helen Skouteris, Karin Leder, for the COVIC-HA investigator group, Peter A. Cameron, Andrew Forbes, Kelsey Grantham, Carol L. Hodgson, Peter Hunter, Jessica Kasza, Philip L. Russo, Malcolm Sim, Kasha Singh, Karen L. Smith, Rhonda L. Stuart, Helena J. Teede, James M. Trauer, Andrew Udy, Sophia Zoungas

**Affiliations:** 1 School of Public Health and Preventive Medicine, Monash University, 553 St Kilda Road, Melbourne, VIC 3004 Australia; 2 Alfred Health, Melbourne, VIC Australia; 3 Royal Melbourne Hospital, Melbourne, VIC Australia

**Keywords:** COVID-19, Occupational health, Healthcare workers, Workplace responses, Perceptions

## Abstract

**Background:**

The COVID-19 pandemic has profoundly impacted individuals, society, and healthcare organisations worldwide. Recent international research suggests that concerns, needs, and experiences of healthcare workers (HCWs) have evolved throughout the pandemic. This longitudinal qualitative study explored the evolving views and experiences of Victorian healthcare workers (HCWs) and organisational key personnel during the coronavirus disease (COVID-19) pandemic.

**Methods:**

We recruited participants from the Coronavirus in Victorian Health and Aged care workers (COVIC-HA) study cohort. We conducted two rounds of semi-structured interviews with HCWs and organisational key personnel from three different healthcare settings (hospital, aged care and primary care) in Victoria, Australia, in May-July 2021 and May-July 2022. Data were analysed thematically using trajectory and recurrent cross-sectional approaches, guided by a temporal change framework.

**Results:**

Twelve HCWs and five key personnel from various professional roles participated in interviews at both timepoints. Expected themes derived from mid-2021 interviews (navigating uncertainty, maintaining service delivery, and addressing staff needs) evolved over time. Concerns shifted from personal health and safety to workforce pressures, contributing to HCW burnout and fatigue and ongoing mental health support needs. New themes emerged from mid-2022 interviews, including managing ongoing COVID-19 impacts and supporting the healthcare workforce into the future. Clear and consistent communication, stable guidelines and forward-looking organisational responses were considered crucial.

**Conclusions:**

Our longitudinal qualitative study highlighted the evolving impact of the COVID-19 pandemic on HCWs’ perceptions, health and wellbeing and uncovered long-term sector vulnerabilities. Analysing HCW experiences and key personnel insights over time and across different pandemic phases provided crucial insights for policymakers to protect the healthcare workforce. Findings emphasise the need for proactive strategies that prioritise HCWs’ wellbeing and workforce sustainability. Policy makers must invest in HCW health and wellbeing initiatives alongside healthcare system improvements to ensure resilience and capacity to meet future challenges.

**Trial registration:**

This study was approved through the Victorian Streamlined Ethical Review Process (SERP: Project Number 68,086) and registered with ANZCTR (ACTRN12621000533897) on 6 May 2021.

**Supplementary Information:**

The online version contains supplementary material available at 10.1186/s12913-024-11067-z.

## Background

Despite variability in the timing and magnitude of cases, hospitalisations, and deaths the COVID-19 pandemic has affected individuals, society, and healthcare organisations globally. In Australia, strict border closures and controversial but effective public health measures kept case numbers low throughout most of 2020 and 2021 [1], but in late 2021 and early 2022 Australia experienced a surge in COVID-19 cases, peaking at over 4000 daily cases per million people in January 2022 [2]. This burdened healthcare workers (HCWs) and the healthcare system.

HCWs have been at the forefront of the pandemic response. Australian research has highlighted the significant psychological toll of the pandemic on HCWs, including high levels of depression, anxiety, post-traumatic stress and burnout [3, 4]. Various pandemic-related factors, such as heightened uncertainty, personal protective equipment (PPE) shortages, communication challenges and inadequate psychological support, contributed to negative HCW experiences [5–8]. Longitudinal research is beginning to solidify our understanding of differential adverse psychological impacts according to pandemic circumstances [9–15], although few studies have used qualitative methods to capture the evolving nature of HCWs concerns, needs, and experiences during different pandemic phases [16]. Moreover, few studies have incorporated perspectives from personnel involved in healthcare organisation responses, which is crucial for understanding the broader context and impact at both individual and organisational levels [7]. 

We adopted a longitudinal qualitative approach to examine evolving views and experiences of Victorian HCWs and key personnel from their healthcare organisations in response to the COVID-19 pandemic.

## Methods

### Study design

This longitudinal qualitative study is part of the Coronavirus in Victorian Healthcare and Aged care workers (COVIC-HA) cohort study, which has investigated impacts of the COVID-19 pandemic on Victorian HCWs and healthcare organisations over time [4, 7, 15]. We report this study in line with the consolidated criteria for reporting of qualitative research (COREQ) and refer readers to the COREQ checklist in the Supplementary material for further details [17]. We interviewed the same participants at two timepoints: May-July 2021 (mid-2021) and May-July 2022 (mid-2022) [18]. Methods and key findings from mid-2021 interviews have been reported previously [7]. 

### Recruitment

We recruited HCWs from the COVIC-HA study cohort, which comprised over 1600 HCWs from hospital, ambulance services, and primary and aged care settings in Victoria, Australia [15]. HCWs included patient-facing and non-clinical staff. Key personnel were senior staff from organisations participating in the COVIC-HA study, nominated for their in-depth knowledge of their organisation’s pandemic response. Some key personnel (e.g., general practice owners) held dual responsibilities as both HCWs and managers and some HCW interview participants held management roles [7]. We aimed to capture a diverse range of experiences from across the healthcare sector. We invited participants of various ages, genders, professions, healthcare settings and levels of COVID-19 exposure (e.g., infection, furlough) to participate. We used purposive sampling to ensure diversity across organisations and healthcare settings. Only those who participated in an interview in mid-2021, comprising of twenty-eight HCWs and twenty-one key personnel, were eligible for follow-up interviews. Consent to be contacted for follow-up interviews was obtained during interviews in mid-2021. Participants were contacted by the research team (SC or OE) to arrange an interview.

### Data collection

Mid-2022 semi-structured interview data were collected by 4 researchers (2 female, 2 male) remotely via Zoom between 11th May and 28th July 2022, using the same methods employed in mid-2021 [7]. Interview guides (Supplementary material) were flexible, with planned topics based on key themes identified from mid-2021 interviews and questions focused on changes over time [4, 7, 15]. Before interviews, interviewers reviewed summaries from each participant’s mid-2021 interview and were encouraged to revisit topics from earlier discussions to explore temporal changes.

### Analysis

We used a reflexive thematic analysis approach following the methodology established by Braun and Clarke [19], adopting an essentialist/realist epistemological stance [20]. We analysed data in a manner that respected and expressed the subjectivity of participant’s accounts, while recognising and embracing the reflexive influence of researchers’ interpretations. Drawing on our backgrounds in public health, psychology and healthcare, we brought firsthand perspectives and understandings of individual experiences, psychological impacts, organisational factors, and broader health implications to our analysis. These insights guided us in interpreting HCWs’ narratives and identifying key themes for practice and policy. We employed a trajectory approach to explore changes in individual experiences and a recurrent cross-sectional approach to explore themes and changes in participants as a whole [21, 22]. Research questions were guided by Saldana’s framework for analysing change through time and included ‘what themes improved, worsened, ceased, emerged or remained constant through time?’, ‘what are the dynamics of participant changes through time?’, and ‘what contextual or intervening conditions appear to influence and affect individual changes through time [23]? . Data from HCW and key personnel interviews were combined to incorporate both the firsthand experiences of HCWs, and the broader context and organisational perspectives provided by key personnel. The initial analysis was conducted by OE (BPH, public health) under the supervision of SLM (PhD, public health). OE immersed himself in the data by actively listening to interview recordings and reviewing participants’ mid-2021 and mid-2022 interview notes and transcripts. OE then crafted individual participant summaries to summarise key experiences, perceptions, and longitudinal changes. OE then uploaded interview transcripts and notes to NVivo software (v.20) and coded them using a combination of deductive and inductive approaches. Deductive coding focused on alignment with themes from analysis of mid-2021 interviews (expected themes) [7]. OE and SLM established a framework to examine evolution of expected themes over time and identify whether new themes from mid-2022 data were present in mid-2021. A primarily inductive approach was used to identify new themes in mid-2022 data. OE and SLM met regularly, with periodic engagement of the wider research team (including JF, PhD, psychology; MK, PhD, psychology; SC, Grad Dip Psych; HK, PhD, occupational health; GR, PhD, health services; HS, PhD, health and wellbeing; KL, PhD, medicine), to discuss and refine topics and themes. We selected one illustrative case study from each healthcare sector, changing all identifying details (Table 1). When reporting findings, we used the term ‘participants’ to describe patterns that emerged consistently across interviews with both HCWs and key personnel.

**Table 1:** Illustrative case studies from each healthcare sector (names are pseudonyms)

**Primary care**: Jamie, a suburban GP in Melbourne, consistently felt let down by the lack of guidance from government agencies (e.g., Primary Health Networks and Department of Health) around “handling of the pandemic” in the primary care sector. They felt that government rhetoric claimed “everything was hunky-dory” on paper, while in practice “it was a bit different.” Initially, they were frustrated by agencies touting positive messages about the vaccine rollout, while “cutting away basic services from general practice”. In 2022, Jamie still believed their practice had to “find [their] own way” amid new “complex” challenges such as the “rise in influenza A…in combination with COVID-19” with little to no support.
**Aged care**: Morgan is an aged care worker whose experience of working during the COVID-19 pandemic improved due to changes in their work environment. In 2021, Morgan described working with a *“constant fear of catching the virus”* when they were redeployed to work in a COVID-19 outbreak facility. There, they and their colleagues felt the burden of being *“stuck in the same area for 12 hours”* and witnessed death as *“nearly an everyday occurrence.”* Morgan’s frustration grew when they acquired COVID-19 at work, despite strictly adhering to infection prevention and control protocols. Despite feeling they will *“always have that fear of recatching”* the virus, over time Morgan felt more *“relaxed”* and was better prepared to manage COVID-19 infections. Upon eventually returning to their usual facility, Morgan found *“life a whole lot better”* and *“enjoyed”* work again. Nevertheless, they were concerned about witnessing their colleagues leave the industry and advocated for greater accessibility and more responsive psychosocial support in the workplace.
**Hospital**: Taylor, a senior representative at a large Victorian hospital service, emphasised the need for organisational adaptation to address staff’s evolving needs. While Taylor’s initial focus in the pandemic was ensuring staff felt *“safe to come to work”*, by 2022, they recognised that systemic issues in the working environment were of greater concern to their team. Taylor was *“not surprised”* that staff were considering leaving the industry, perceiving that the system wasn’t meeting workers needs or enticing them to stay. Taylor witnessed the strain on staff amid staff shortages and increased sick leave and advocated for *“clear short- to medium-term planning…[to] manage this workload moving forward”.* They believed that *“suboptimal physical environments”* was the *“number one under-recognised problem,” and “investing in people”* through enhanced professional development opportunities was vital.

## Results

Of twenty-eight HCWs interviewed in mid-2021, twenty consented to be contacted for a follow-up interview, however eight were unavailable or unreachable. Among twenty-one key personnel interviewed in mid-2021, eight consented to be contacted for a follow-up interview, but three were unreachable. Twelve HCWs and five key personnel participated in interviews at both timepoints. HCWs included seven hospital workers, three aged care and two primary care workers. Half were female and most were aged between 40 and 59 years. Professional roles varied: three nurses, three doctors, one Allied Health worker, two Personal Care Assistants (PCAs) and three in support and administrative roles. Key personnel comprised three participants from hospital settings, and one each from primary and aged care settings. Professional roles covered medical executive, practice management, clinical governance, and infection control functions; three were female. Mid-2021 interviews averaged 35 min; mid-2022 interviews averaged 51 min (range: 28–94 min).

### Evolution of expected themes

The three major themes and nine sub-themes identified in mid-2021 interview data (expected themes) are summarised in Table 2 and have been described in detail elsewhere [7]. Table 3 illustrates how expected themes changed over the course of the 2 interviews.

**Table 2:** Themes and subthemes derived from 2021 interviews (“Expected themes”) with illustrative quotes

Theme	Subtheme	Illustrative quotation
**Navigating a changing and uncertain environment**	Pandemic (un)preparedness	*I thought that it was lucky for Australia to have that kind of lag time…. I was very upset when things could have been done, which weren’t. …. (HCW, primary care, female, aged 50–59 years)*
	Clear and consistent communication	*It was quite difficult to keep up with what was going on, and make sure that everybody was operating using the most current information. (KP, aged care, female)*
	Active engagement between decision makers and workers	*A lot of decisions were being made by executives that were sitting at home and didn’t have an idea of what things actually looked like on the ground. (HCW, doctor, hospital, male, aged 40–49 years)*
**Maintaining service delivery during a pandemic**	Greater physical and mental demands of work	*I’m normally a very caring person but I feel I no longer care and have minimal job satisfaction due to burnout and fatigue. (HCW, paramedic, ambulance, male, aged 30–39 years)*
	Sustaining the response	*As outbreaks have progressed … complacency creeps in. Staff become tired. Staff become sick of having to put on PPE. (KP, aged care, female)*
	Resourcing and logistics	*“To run COVID vaccinations from the general practice, at the same time as flu vaccinations… became too difficult. We just didn’t have enough staff to do that. (KP, primary care, female)*
**Meeting the psychological and safety needs of staff**	Infection prevention control, training, and guidance	*Before COVID, we assumed that PPE was a simple thing, that staff were using it all the time, … but what we discovered is that they really didn’t have the skills. (KP, hospital, female)*
	Investment in mental health and wellbeing services	*[We need] an investment into the psychological wellbeing of staff now and into the future. (HCW, doctor, hospital, male, aged 40–49 years).*
	Acknowledgement and recognition	*Even though it was hard work, it was nice to be appreciated, (HCW, nurse, primary care, female, aged 30–39 years).*

*HCW *Healthcare worker, *KP *Key personnel

**Table 3:** Evolution of expected themes and subthemes

	Mid-2021 (May – July 2021)	Mid-2022 (May – July 2022)
**Theme: Navigating a changing and uncertain environment**		
Pandemic (un)preparedness	Anxiety and uncertainty around COVID-19	Acceptance of COVID-19 the “new normal”
	Wasted time and effort preparing for “doomsday scenarios”	Worst-case scenario planning came to fruition
	Existing structures unsuitable for pandemic response	Adaptation of structures to suit pandemic demands
	Proactive and forward-thinking responses needed	
Clear and consistent communication	Excessive communication and policy changes causing staff confusion	More consistent policy environment with timely and streamlined communication
	Lack of transparency around communicating workplace COVID-19 infections	Improved communication but heightened uncertainty around COVID-19 infection acquisition
Active engagement between decision makers and workers	Limited HCW engagement	Improved ways for HCWs to voice concerns
	Remote leadership fostering an us-and-them dynamic. Visible, accessible leaders valued	On-site leaders promoting collaboration and reducing “top-down” decisions
**Theme: Maintaining service delivery during a pandemic**		
Greater physical and mental demands of work	Burden of enhanced infection control measures	Staff struggling to disconnect from work
	Emotional toll of witnessing death and separation	Growing departure of skilled workers
Sustaining the response	Stretched and fatigued workforce	Increasingly fatigued and burnt-out workforce
	Staff movement restrictions	Catastrophic staff shortages
	Frequent HCW furlough and loss of student workers	Increased burden on senior staff
Resourcing and logistics	Fluctuating service demands and mandates (e.g. reductions in elective surgery)	Persistent high COVID-19 caseload and service demands
	Improved digital integration in the workplace	
**Theme: Meeting the psychological and safety needs of staff**		
Infection prevention control, training, and guidance	HCWs worried about bringing COVID-19 home from work	HCWs worried about bringing COVID-19 to work
	COVID-19 wards perceived as risk areas	Non-COVID-19 wards and non-clinical spaces perceived as risk areas
	Confidence in PPE undermined by changing policies	Greater confidence in PPE
	Hypervigilance around infection control	Growing complacency around infection control
Investment in mental health and wellbeing services	Morale and team building initiatives highly valued by staff	Accessible and approachable managers highly valued
	Greater investment in mental health and wellbeing services	
Acknowledgement and recognition	Genuine expressions of acknowledgement valued	Acknowledgement alone is insufficient to address HCW needs
	Advocacy for improvements in working environment and financial remuneration	

*PPE *Personal protective equipment

### Navigating a changing and uncertain environment

In mid-2021 interviews, HCWs and key personnel recounted widespread anxiety and uncertainty at pandemic’s onset. Prominent concerns included fear of infection and lack of pandemic preparedness, which were amplified by inconsistent policies, communication challenges and slow vaccination rollout. HCWs reported limited transparency around workplace COVID-19 infections, and a perception that leaders were not facing the same risks as frontline staff fostered an us-versus-them dynamic.

During mid-2022 interviews, participants expressed collective acceptance of COVID-19 as the *“new normal” (HCW, nurse, hospital, 50-59yrs).* Overall, HCWs were *“less anxious and fearful” (KP, aged-care, female)* and more *“relaxed” (HCW, PCA, aged-care, 40-49yrs)* in exposure situations, with factors contributing to this shift including improved familiarity with and confidence in PPE, higher community vaccination rates and the availability of new treatments. Participants felt that both individuals and organisations had learned from previous experiences and adapted their responses. Hospital and aged care participants indicated that *“worst case scenario” (HCW, nurse, hospital, female, 50-59yrs)* planning “*came to fruition” (KP, aged care, female)* as COVID-19 case numbers increased.

However, some HCWs felt that organisational responses could have been more “*proactive and forward-thinking” (HCW, other, hospital, male, 50-59yrs)*. Additionally, some were unconvinced that their organisation knew *“what the plan [was] going forwards.” (HCW, allied health, hospital, female, 40-49yrs)*. Nevertheless, several participants viewed their pandemic experiences as valuable lessons to be carried forward in readiness for future pandemic waves or other public health emergencies.

By mid-2022, there was a sense that *“communication [was] definitely better” (HCW, nurse, hospital, female, 40-49yrs)*, with fewer concerns around clarity and consistency. Greater familiarity with COVID-19 led to more stable case and outbreak management protocols, resulting in less frequent and more timely communications. This alleviated pressure and reduced confusion among HCWs. Effective management teams played a key role in facilitating information dissemination and boosting confidence. While transparency concerns around worker infections dwindled, and COVID-19 infection was no longer viewed as a *“dirty little secret” (HCW, nurse, hospital, female, 50-59yrs)*, increased transmission and reduced contract tracing efforts left HCWs grappling with heightened uncertainty around infection acquisition and transmission.

In mid-2022, HCWs described improved ways to voice concerns, fostering active engagement between decision-makers and workers. They appreciated expert- and CEO-led question and answer sessions and staff forums, and anonymous feedback mechanisms such as surveys. As senior staff resumed face-to-face work arrangements, instances of *“top-down decision-making” (HCW, doctor, primary care, male, 50-59yrs)* decreased, leading to enhanced collaboration. However, some HCWs still felt their concerns were *“falling on deaf ears” (HCW, other, hospital, male, 50-59yrs)*, leading to frustration and a sense of being disregarded. Participants also highlighted a lack of consultation in government-level decision-making, especially within the primary care sector.

### Maintaining service delivery during a pandemic

In mid-2021, participants described acute challenges arising from rapid workforce mobilisation to meet pandemic demands. Workers faced increased physical and mental pressures due to enhanced infection control measures and the emotional toll of witnessing death and separation. Rapid mobilisation led to expanded digital technology use and infrastructure changes, but fluctuating service demands and mandates introduced challenges, especially in the private sector. Additionally, concerns arose about sustaining the response as initial enthusiasm waned and organisations grappled with workforce shortages.

By mid-2022, HCWs and key personnel felt that the situation was “*getting harder the longer it goes.” (KP, hospital, female)* with a growing acceptance that there was *“no real end in sight” (HCW, allied health, hospital, female, 40-49yrs)*. HCWs felt *“in it all the time” (HCW, doctor, primary-care, male, 50-59yrs)* and struggled to disconnect, affecting their resilience and motivation to cover shifts or work extra hours. The sustained demands of working in a pandemic environment took a toll, leading to widespread burnout and fatigue. The healthcare system was described as *“broken” (KP, hospital, female)*, with a “*drained and exhausted”* (*HCW, nurse, hospital, female, 40-49yrs)* workforce.

Widespread workforce shortages were exacerbated by rising community transmission, mental and physical stress, and at-home caring responsibilities. Nurses were seen as shouldering a disproportionate burden, with one HCW describing their workforce as being *“decimated” (HCW, nurse, aged-care, male, 30-39yrs).* HCWs, particularly in hospital and aged care settings, expressed concerns about delivering usual standards of patient care. In primary care, participants cited examples of increased patient demand and a shortage of doctors resulting in extended patient wait times.

Amid widespread shortages, concerns grew about the ongoing departure of skilled workers. Some called for a re-evaluation of redeployment practices, advocating to backfill workers in specialised roles. Participants highlighted that junior staff, whose education had already been disrupted, found themselves in increasingly complex roles without adequate preparation. Meanwhile, senior staff faced additional burdens including adjusting to new command structures, taking on unfamiliar roles, and the expectation of being continuously available (whether on-site or via telephone) and accessible to staff in need.

Workers who observed colleagues leaving the profession noted that some left due to impending retirement or better job offers, while others struggled with the *“mental trauma” (KP, hospital, female)* associated with their work. Organisational efforts to address staff shortages had mixed reviews. Reliance on external workers, such as students, agency staff and redeployed personnel, raised concerns about expertise and accountability and was seen as a short-term solution. Longer-term solutions, such as partnering with a local Technical and Further Education (TAFE) provider to offer a ‘train-while-you-work’ program, were commended.

The expansion of digital health technologies in healthcare emerged as a pandemic silver living, despite challenges in upskilling workers and upgrading systems to meet new demands. Enhanced digital integration in the hospital settings was seen to facilitate remote work, service accessibility and efficiency throughout 2021 and 2022. In contrast, participants from the primary care sector encountered logistical challenges in delivering telehealth and had limited technical support. The return of in-person consultations was seen to reduce the threat of *“shut down[s]” (KP, primary care, female)* in primary care with practice owners and managers growing more optimistic about their *“practice’s ongoing viability” (HCW, doctor, primary-care, male, 50-59yrs)*.

### Meeting the psychological and safety needs of staff

In mid-2021, participants stressed the importance of addressing staff’s psychological and safety needs. Although staff valued the swift improvement in infection control training and guidance, they often lacked confidence in the reliability of PPE due to frequent policy changes. There was consensus on the need for greater investment in wellbeing and mental health services as staff tackled increased physical and emotional burdens. While HCWs welcomed recognition of their efforts, concerns arose when such recognition was perceived as insincere or inadequate.

In mid-2022, HCWs continued to value infection control training and guidance, even though their working environment *“was very different” (HCW, doctor, hospital, male, 40-49yrs)*. While they remained vigilant, the availability of fit-testing programs, higher-quality PPE and vaccination instilled a sense of confidence among staff, making them feel *“pretty bulletproof” (HCW, allied health, hospital, female, 40-49yrs)* when adhering to infection control standards. Enhanced familiarity with COVID-19 management empowered more wards and facilities to independently *“manage their own situations” (HCW, allied health, hospital, female, 40-49yrs).*

However, participants noted growing complacency around correct infection control practices, particularly regarding maintaining *“standards and expectations” (HCW, nurse, aged-care, male, 30-39yrs)* of PPE adherence. While standards remained high when dealing with COVID-positive patients, it was observed that staff were *“less vigilant” (KP, hospital, male)* in areas like tea-rooms and general wards. Heightened community transmission and greater uncertainty around infection acquisition shifted concerns “*from I’m going to take COVID home, to I’m going to take COVID to work.” (HCW, nurse, hospital, female, 50-59yrs)*. Coupled with reduced contract tracing efforts, this was seen to compromise outbreak management, particularly in aged-care settings.

In mid-2022, as burnout and exhaustion became more prevalent, participants reiterated the need for greater investment in wellbeing and mental health. HCWs discussed ways to foster positive team environments, such as using humour and organising regular workplace activities, which were positively received. However, key personnel often described how organisational efforts to prioritise staff wellbeing were frequently hindered by a mismatch between demand and availability, as well as time constraints due to heightened workloads and staff shortages.

Hospital and aged care workers continued to report more formal support programs, such as Employee Assistance Programs (EAPs) compared to those in primary care. However, concerns persisted regarding the availability of and access to these services. HCWs believed that delayed access to support drastically reduced its value and that services could be more effective if they were integrated *“into the fabric” (HCW, doctor, hospital, male, 40-49yrs, R2)* of healthcare organisations. Participant suggestions for fostering a more proactive and supportive workplace wellbeing culture included increased promotion of services, the use of *“on-site psychologists”* (*HCW, PCA, aged-care, male, 40-49yrs)* and tailoring services to individual needs and specific fields of work. Accessible and approachable managers who made themselves available for discussions and debriefing were especially valued and perceived to have positive flow-on effects to HCW wellbeing.

Acknowledgement and recognition of staff remained a prominent sub-theme in mid-2022 interviews. Some HCWs expressed a sense of being *“cast aside” (HCW, doctor, hospital, male, 40-49yrs)* by this stage of the pandemic, citing minimal improvements in their working environment and inconsistent media rhetoric regarding their efforts.

HCWs viewed increased financial remunerations as *“paltry offerings” (HCW, doctor, hospital, male, 40-49yrs)*, with a growing demand for better allowances. Key personnel echoed these sentiments, emphasising that remuneration should be proportionate to the demanding work undertaken by healthcare staff. In the private sector, staff felt *“undervalued” (KP, hospital, male)* when they didn’t receive the same incentives as their public sector colleagues. The pandemic was also seen to exacerbate existing disparities in recognising the work of non-clinical staff.

Key personnel across all sectors emphasised the importance of striking a balance between genuine expressions of acknowledgement and actions that *“appear insincere or inappropriate” (KP, hospital, male).* HCWs continued to value flexible working arrangements, meal provisions and initiatives that enabled them to get out of their clinical spaces, such as ice cream and coffee vans. Nevertheless, there was a consistent feeling that more was needed to truly motivate workers to stay in their roles.

#### New themes

Analysis of mid-2022 interview data yielded two new themes each with two sub-themes (Table 4). The theme ‘*Managing ongoing COVID impacts’* reflected the ongoing COVID-19 management challenges faced by HCWs and organisations amid the relaxation of public health restrictions and waning public anxiety. Likewise, the theme ‘*Strengthening and supporting the workforce into the future’* emerged in response to the sustained high demand for healthcare services and persistent HCW shortages.

**Table 4:** New themes and subthemes emerging from mid-2022 interviews with illustrative quotations

Theme	Subtheme	Illustrative quotation
**Managing ongoing COVID impacts**	Public perception versus healthcare realities	*“The rest of the world is pretending like COVID doesn’t exist, and yet we’re about to open another COVID ward.” (HCW, nurse, hospital, female, 50-59yrs)*
	Disregard for ongoing mitigation measures	*“Our policy is that you cannot come into [the facility] unless you do a [RAT], and I’ve had to argue with family members who don’t want to do it. Well, I’m sorry you’re a selfish idiot…your inconvenience for like 10 s and a 15-minute wait could be the difference between people dying and not dying.” (HCW, nurse, aged-care, male, 30-39yrs)*
**Strengthening and supporting the workforce into the future**	Long-term investment in staff	*“We need to be training these people, and not only invest in their training, but then properly support them, otherwise they’ll just leave.” (HCW, doctor, hospital, male, 40-49yrs)*
	Addressing systemic issues for a stronger healthcare workforce	*“If we felt that the system was being fixed. If we had a better working environment, the pay actually isn’t as important.” (HCW, doctor, hospital, male, 40-49yrs)* *“I’d love to give people their accrued annual leave…the problem at the moment is with the workforce shortages, it’s actually quite hard to take the break” (KP, hospital, male)*

#### Managing ongoing COVID impacts

Participants described a discrepancy between public perceptions and healthcare realities. COVID-19 had seemingly become *“yesterday’s news” (KP, hospital, male)* in the eyes of the public but remained a *“massive strain” (HCW, doctor, hospital, male, 40-49yrs)* for many HCWs and organisations. Many HCWs believed that the *“desire to return to normal” (HCW, doctor, hospital, male, 40-49yrs)* had taken precedence over community safety and felt that the consequences of relaxed community infection control strategies on the healthcare system were *“just completely ignored” (HCW, allied health, hospital, female, 40-49yrs)*. Participants also felt that the severity and consequences of COVID-19 infections were under-appreciated by the public and downplayed by governments, with these attitudes reflected in workplace policies.

Concern was raised over the lack of awareness and clinical understanding of *“long-term post-COVID symptoms” (HCW, other, hospital, female, 50-59yrs)*. This was seen as detrimental to workplace policies concerning return of staff to work, with many feeling that organisations were rushing workers *“straight back into it” (HCW, doctor, hospital, male, 40-49yrs)*, rather than allowing them the necessary time to recover.

Across all settings, HCWs and key personnel grappled with community disregard for COVID-19 mitigation measures in healthcare settings as community restrictions eased, with reports of verbal abuse and hostile confrontations.

#### Strengthening and supporting the workforce into the future

While participants recognised that organisations have met the *“basic human needs” (KP, hospital, male)* of staff, a recurring theme emphasised the necessity of long-term investment in staff to *“attract and retain” (KP, hospital, male)* HCWs into the future. Amidst sustained workforce pressures, workplaces faced the challenge of retaining existing and attracting new staff. HCWs saw a need to *“appreciate [staff] potential and… empower [their] professional growth” (HCW, other, hospital, female, 50-59yrs)*. Suggestions included strengthening onboarding for new staff and providing opportunities for “*ongoing professional development” (HCW, nurse, hospital, female, 50-59yrs)*, including for roles that traditionally lack such options.

Additionally, participants wanted more sustainable plans to address operational and logistical issues, in particular workforce shortages. Initiatives like increased wages were appreciated but were seen as doing little to address ongoing problems and improve *“the life of healthcare workers” (KP, hospital, male).* To ensure staff want to *“turn up every day” (KP, hospital, female) and “strengthen the workforce moving forward” (HCW, other, hospital, female, 50-59yrs)*, suggestions included prioritising leave entitlements to provide ample *“downtime” (HCW, doctor, primary care, male, 40-49yrs)*, investing in better working conditions and physical environments, and long-term planning to address workforce availability.

## Discussion

The impact of the COVID-19 pandemic on the physical and psychological health of HCWs has evolved but persisted [3, 24]. Our findings indicate a shift in HCWs’ concerns and needs over time, aligning with longitudinal qualitative research from the UK [16]. The heightened fear and anxiety HCWs faced in mid-2021 from the rapidly changing and uncertain pandemic landscape, and the fears of bringing COVID-19 home to their families [25, 26] were replaced by an adaption to COVID as the “new norm” by mid-2022, with stable policies, transparent communication, and increased consultation bolstering HCWs sense of security and empowerment. However, concerns of widespread exhaustion, fatigue, and risks of bringing COVID-19 to the workplace grew and PPE adherence declined [27]. This underscores the challenges of maintaining effective infection prevention practices even in healthcare settings [28]. Especially during a pandemic, HCWs emphasised the importance of offering flexible, specialised and tailored mental health services on-site [29, 30]. 

In mid-2022, HCWs and key personnel related that high COVID-19 case numbers and increasing staff turnover had exacerbated workforce shortages. The appeal of retirement, better job offers in terms of work and/or pay, acute staff shortages and rising fatigue and burnout have contributed to more HCWs considering leaving their profession, especially among nurses for whom high pre-pandemic rates of turnover were further exacerbated [31]. Consequently, addressing shortfalls within this critical healthcare profession is imperative well beyond the scope of the COVID-19 pandemic [32]. Student placement strategies were seen as an effective approach to combat workforce shortages in the aged care sector, and have shown effectiveness in other health settings as well [33]. As observed in previous public health crises, HCWs emphasised the need for better pay [34, 35], although this was viewed as an inadequate short-term fix. Sustainable, long-term solutions focused on the well-being and needs of workers are needed to combat shortages and strengthen the healthcare workforce [36], including improved onboarding practices, improving working conditions and the physical work environment, and expanded professional development opportunities.

HCWs perceive a significant gap between public perceptions of COVID-19 and the ongoing strain on healthcare systems, potentially resulting in workplace violence [37]. Staff safety, including the enforcement of anti-abuse and anti-aggression policies, should remain a top organisational priority, and visitors should respect and adhere to these policies [38]. Organisational commitment, increasing on-site security, improved reporting of incidents and increasing staff-to-patient ratios may help alleviate HCW concerns [37]. 

Our study’s major strength lies in its longitudinal qualitative design, which actively engaged HCWs and key personnel from various healthcare sectors. This emerging approach in health research recognises the fluid nature of human experiences, offering a valuable means of exploring changes over time and identifying critical processes driving change [39, 40]. By including both HCW and key personnel perspectives, we gained a clearer understanding of the impact of the COVID-19 pandemic on individuals and organisations. Critically, our in-depth analysis occurred within the context of two different pandemic phases: one marked by restrictive measures and uncertainty with fewer cases (mid-2021), and another characterised by high case numbers, relaxed policies and changed public perception (mid-2022). This allowed us to explore how HCW perceptions evolved and organisational responses varied across different pandemic stages. Our iterative and participative approach ensures that our findings are meaningful and reflective of real-world experiences. It provides crucial insights for policy makers to protect the healthcare workforce from impacts beyond the immediate scope of the pandemic. We implemented several strategies to enhance trustworthiness of the results, including prolonged engagement with the data, peer debriefing, application of a coding framework, and team consensus on themes. While our findings generally align with prevalent narratives, our scientific documentation of these attitudes can serve as a catalyst for policy changes.

However, our study has some limitations. Recruitment primarily focused on healthcare organisations in South-east Melbourne, Victoria and we were only able to recruit a small follow-up sample, which may limit the transferability of our findings [41]. While efforts were made to ensure credibility of our findings, our observations were confined to two timepoints, 12-months apart. Additional observations over the period of engagement may have enabled a more precise assessment of the impact of pandemic phases on the evolving views and experiences of our study participants.

## Conclusion

The COVID-19 pandemic’s impact on HCWs and organisations has changed over time. While some initial challenges have diminished, concerns of widespread exhaustion, fatigue, burnout and risks of bringing COVID-19 to the workplace emerged and PPE adherence declined. To address these challenges effectively and strengthen the health workforce, organisational responses must adopt a forward-thinking approach incorporating substantial, long-term investments into the physical, mental and wellbeing needs of HCWs. As the healthcare system transitions from an emergency pandemic response to a sustained one, it is imperative that healthcare organisations adapt and cater to the evolving, long-term needs of their staff. Urgent action for ongoing funding and system-wide reforms for the healthcare sector is essential, not only to safeguard the workforce but also to maintain the quality-of-care delivery for the community. In this ever-evolving healthcare landscape, resilience, adaptation, and support for HCWs are central to ensuring a robust and sustainable healthcare system for the future.

### Supplementary Information

Supplementary Material 1.

## Data Availability

The datasets used and/or analysed during the current study are available from the corresponding author on reasonable request.
