# Supplementary material for: A longitudinal qualitative exploration of Victorian healthcare workers’ and organisations’ evolving views and experiences during COVID-19
Source: BMC Health Serv Res. 2024 May 7;24:596. doi: 10.1186/s12913-024-11067-z (PMC11077789; doi:10.1186/s12913-024-11067-z)
Supplement: Supplementary file 1 — Supplementary Material 1. [file 12913_2024_11067_MOESM1_ESM.docx]

## Supplementary Table 1: Consolidated Criteria for Reporting of Qualitative Research (COREQ) Checklist

|  | **Item** | **Guide questions/description** | **Response** |
| --- | --- | --- | --- |
| ***Domain 1: Research team and reflexivity*** | | | |
| *Personal Characteristics* | *Interviewer/facilitator* | *Which author/s conducted the interview or focus group?* | Sarah McGuinness  Owen Eades  Sharon Clifford  Riki Lane |
|  | *Credentials* | *What were the researcher’s credentials? E.g. PhD, MD* | Sarah McGuinness – MBBS, BMedSc, DTMH, MPH&TM, FRACP, FACTM, PhD  Owen Eades – BPH  Sharon Clifford – BSc (Hons), BA, MC, Grad.Dip.Psych  Riki Lane – BA/BSc (Hons), PhD |
|  | *Occupation* | *What was their occupation at the time of the study?* | Sarah McGuinness – Infectious Diseases Physician, Lecturer & Researcher  Owen Eades – Project/Research Officer  Sharon Clifford – Research Project Manager  Riki Lane – Research Fellow |
|  | *Gender* | *Was the researcher male or female?* | Male and female |
|  | *Experience and training* | *What experience or training did the researcher have?* | Sarah McGuinness is a clinician and established researcher in public health and infectious disease epidemiology, with expertise in quantitative and qualitative methods.  Owen Eades is an emerging qualitative researcher with experience conducting and coordinating qualitative research.  Sharon Clifford is an experienced research coordinator with skills in qualitative research and long-standing connections in the Victorian primary health care network.  Riki Lane is an experienced qualitative and ethnographic researcher.  Prior to commencement of semi-structured interviews, all interviewers attended an orientation session conducted by Maggie Kirkman (a senior qualitative researcher from the Monash University School of Public Health & Preventative Medicine). The session was recorded and circulated to the interviewing authors, for reference. |
| *Relationship with participants* | *Relationship established* | *Was a relationship established prior to study commencement?* | The study’s Project Officer (Owen Eades) was responsible for communications with healthcare workers (HCWs) and key personnel from the hospital, and aged care streams to arrange interviews and was involved in qualitative interviewing. Sharon Clifford from the Department of General Practice was responsible for communications with HCWs from the primary care stream and also conducted some qualitative interviews with these individuals. |
|  | *Participant knowledge of the interviewer* | *What did the participants know about the researcher? e.g. personal goals, reasons for doing the research* | Participants were provided with a written explanatory statement, which outlined the rationale and objectives of the research. Participants were informed of researchers’ positions and qualifications prior to data collection. |
|  | *Interviewer characteristics* | *What characteristics were reported about the inter viewer/facilitator? e.g. Bias, assumptions, reasons and interests in the research topic* | Participants were informed that their interview would be conducted by an experienced qualitative researcher who was not associated with the organisation that they worked for. Participants were informed that researchers conducting interviews were interested in exploring the broader effects of the COVID-19 pandemic on HCWs over time, and exploring healthcare organisations’ responses to the pandemic from the perspective of HCWs and organisational key personnel across different stages of the pandemic. |
| ***Domain 2: Study design*** | | | |
| *Theoretical framework* | *Methodological orientation and theory* | *What methodological orientation was stated to underpin the study? e.g. grounded theory, discourse analysis, ethnography, phenomenology, content analysis* | This study was part of the broader Coronavirus in Victorian Aged care and Healthcare workers (COVIC-HA) cohort study, which has a longitudinal mixed methods design involving quantitative surveys and semi-structured qualitative interviews. Qualitative analysis of semi-structured interview data followed a reflexive thematic analysis approach according to the method established by Braun & Clarke (as outlined in Braun & Clarke 2019 and on the following website: <https://www.psych.auckland.ac.nz/en/about/thematic-analysis.html>).  Longitudinal qualitative analysis of interview data was guided by Saldana’s framework for analyzing change through time (Saldana 2003), and used a trajectory approach to explore changes in individual experiences and a recurrent cross-sectional approach to explore themes and changes in participants as a whole (Grossoehme & Lipstein 2016). |
| *Participant selection* | *Sampling* | *How were participants selected? e.g. purposive, convenience, consecutive, snowball* | HCWs and key personnel involved in the second round of semi-structured interviews were sampled using convenience techniques from the pool of participants who had completed an interview for the COVIC-HA Study 12 months prior. |
|  | *Method of approach* | *How were participants approached? e.g. face-to-face, telephone, mail, email* | HCWs identified from the pool of participants who completed an interview 12 months prior were invited to express interest participating in a second interview by responding to a Google Form, sent via email. Interested participants were contacted by Owen Eades or Sharon Clifford via phone/email and provided with a detailed explanatory statement and a list of potential interview times. Once a time was confirmed, participants were sent a calendar invite and Zoom video conference link.  Key personnel identified from the pool of participants who completed an interview 12 months prior were contacted individually via email to express interest. Those who expressed interest were contacted to arrange an interview using the approach as described for HCW participants. |
|  | *Sample size* | *How many participants were in the study?* | 12 HCWs and 5 key personnel participated in qualitative interviews. |
|  | *Non-participation* | *How many people refused to participate or dropped out? Reasons?* | 8 HCWs from the original pool of 28 HCW participants in mid-2021 did not express interest participating in a second interview. Of the 20 who had expressed interest, 4 did not respond when contacted to arrange an interview, 3 rescinded interest due to availability, and 1 participant missed their interview appointment and were not able to reschedule. All other HCWs who were approached for interviews consented to participate.  13 of the original pool of 21 key personnel participants in mid-2021 did not express interest participating in a second interview. Of the 8 who had expressed interest, 3 did not respond when contacted to arrange an interview. All other key personnel who were approached for interviews consented to participate. |
| *Setting* | *Setting of data collection* | *Where was the data collected? e.g. home, clinic, workplace* | Interview data was collected via the video-conferencing platform Zoom; individual sessions were recorded with participants’ consent. Participants could choose to join the video conference from their home or workplace as suited them. |
|  | *Presence of non-participants* | *Was anyone else present besides the participants and researchers?* | No |
|  | *Description of sample* | *What are the important characteristics of the sample? e.g. demographic data, date* | For the purposes of the qualitative component of the research, demographic data obtained and reported was age, gender, work stream and work experience, study site location and exposure to COVID-19. |
| *Data collection* | *Focus group guide and interview guide* | *Were questions, prompts, guides provided by the authors? Was it pilot tested?* | Two interview topic guides were developed separately for HCWs and key personnel, with planned topics based on key themes identified from mid-2021 interviews. Each were piloted within the study’s investigator group prior to interview commencement. The topic guides included a series of open-ended questions and prompts but allowed interviewers to alter the sequence of questions or the way in which they were phrased. Participants were not provided with the topic guide prior to interviews. |
|  | *Repeat interviews* | *Were repeat interviews carried out? If yes, how many?* | No |
|  | *Audio/visual recording* | *Did the research use audio or visual recording to collect the data?* | Interviews were conducted via video-conference, with audio recording enabled. A professional transcription service was engaged to transcribe audio recording into word documents. Following transcription, a member of the research team assessed the transcripts against the original recording for accuracy. |
|  | *Field notes* | *Were field notes made during and/or after the interview or focus group?* | Researchers made field notes during interviews. |
|  | *Duration* | *What was the duration of the inter views or focus group?* | Interview duration was dependent upon how much the participant wanted to say. Average interview duration across the 12 HCW and 5 key personnel interviews was 51 minutes with a range of 28-94mins. |
|  | *Data saturation* | *Was data saturation discussed?* | The concept of data saturation doesn't align with the objective of this research, which was to analyse evolving experiences and views of HCWs and key personnel throughout different pandemic stages. Instead of seeking data saturation, we intentionally recruited participants who had been interviewed 12 months prior. These participants represent diverse demographics, professions, sectors, and levels of COVID-19 exposure, ensuring a wide spectrum of views relevant to our research question. |
|  | *Transcripts returned* | *Were transcripts returned to participants for comment and/or correction?* | No |
| ***Domain 3: Analysis and findings*** | | | |
| *Data analysis* | *Number of data coders* | *How many data coders coded the data?* | One |
|  | *Description of the coding tree* | *Did authors provide a description of the coding tree?* | Yes. Table 2 provides an illustration of the changes in themes and sub-themes between mid-2021 and mid-2022 interviews. Table 3 describes the source of themes and sub-themes unique to the mid-2022 data in our research. |
|  | *Derivation of themes* | *Were themes identified in advance or derived from the data?* | Unexpected themes were unique to the mid-2022 interview dataset and were derived inductively from the data following an established methodology stipulated by Braun & Clarke.  Expected themes were identified as themes that were present in the Mid-2021 interview data, and were analysed deductively as stipulated by Braun & Clarke. |
|  | *Software* | *What software, if applicable, was used to manage the data?* | NVivo version 20 |
|  | *Participant checking* | *Did participants provide feedback on the findings?* | No |
| *Reporting* | *Quotations presented* | *Were participant quotations presented to illustrate the themes/findings? Was each quotation identified? e.g. participant number* | Participant quotations from each data source were included in findings. The source of data, profession and study setting of the quoted individual is indicated for each quotation. A unique and de-identified study code was assigned to each participant to identify the source of quotations (e.g. HCWI08 = Healthcare worker participant, interview 8) |
|  | *Data and findings consistent* | *Was there consistency between the data presented and the findings?* | Yes |
|  | *Clarity of major themes* | *Were major themes clearly presented in the findings?* | Yes |
|  | *Clarity of minor themes* | *Is there a description of diverse cases or discussion of minor themes?* | Yes. Themes and their inherent subthemes are reported in this study from a diverse set of participants. |

**Supplementary File 1:** Healthcare worker (HCW) semi-structured interview guide

**Opening (preamble)**

- Thank the participant
- Do I have your permission to begin recording?
- Preamble: This time last year (May-July ’21) we heard from you about your experiences of COVID-19 and perceptions of your organisation’s response – particularly in relation to leadership, the work environment and training & support. At that time, the COVID-19 situation in Victoria was very different; guidelines were changing rapidly, the vaccine rollout was still in its early stages and aggressive public health control strategies (such as lockdowns and border closures) aimed at suppressing cases were in place. Fast forward to now and we’re learning to live with COVID-19. Today, we’re interested in finding out where you’re at now, compared to 12 months ago, and what you would like to see your organisation doing moving forward?
- Do you have any questions after reading the explanatory statement?
- Do you consent to participating in the interview?

**Professional role and impact of COVID-19 on work role/**

Can you tell me a little about your professional role and how COVID-19 has impacted on your work and your role in the past 12 months?

**Challenges and managing them**

Can you tell me about any challenges you’ve faced in the last 12 months, and how these compare to challenges you faced earlier in the pandemic?

*Potential prompts (if needed):*

- At work? At home? Financial? Feelings of isolation? Separation from family?
- What helped? What didn’t help?
- What did your organisation do to support you? What else could they have done?

**COVID exposures**

if your attitude to COVID-19 risks and exposures has changed in the last 12 months, can you tell me about these changes? At work / outside of work? Caring for cases? Personal safety? Fear of exposure?

- If isolated/quarantined – experiences? Feelings on returning to work?
- What did your organisation do to support you? What else could it have done?

**Organisational responses**

Can you tell me about some thoughts about how the leaders or senior personnel within your organisation have responded to the ongoing impacts of COVID over the past 12 months?

- What did your manager/leaders do that you appreciated?
- Was there anything they did that you did not appreciate?

Can you tell me about some thoughts about any changes to your workload or workflow in response to COVID-19 in the last 12 months?

- Staffing, resourcing, infrastructure, workflow etc
- How do you feel about these changes?

What support has your organisation offered that has helped you in the last 12 months?

- Training? COVID-19 test access? Mental health and wellbeing?
- Do you think these supports will be sustained into the future?
- How did this help? Did you feel it was adequate? Are you able to access it easily?

Can you talk about any opportunities you’ve had to suggest improvements in your organisation’s response and what came about from this?

Our latest round of survey findings indicates that 50% of HCWs who have completed the survey so far, have considered leaving their profession as a result of COVID-19, what are your thoughts on this?

Looking forward, what additional workplace supports would be helpful?

Is there anything else you’d like to say about your experiences of COVID-19 or your perspective on how your organisation has managed it?

**Closing**

- Thank you for your valuable contribution
- If you think of something you wish you’d said, please email [covicha@monash.edu](mailto:covicha@monash.edu). It will be added to your transcript
- Would you be willing to be contacted for another interview in the future?
- Collect demographic details ie. Role, age, gender & work experience (if not already stated)
- Where would you like us to send your $50 digital gift voucher?

**Supplementary File 2:** Key informant semi-structured interview guide

**Opening (preamble)**

- Thank the participant
- Do I have your permission to begin recording?
- Preamble: This time last year (May-July ’21) we heard from you about your experiences of COVID-19 and perceptions of your organisation’s response – particularly in relation to leadership, the work environment and training & support. At that time, the COVID-19 situation in Victoria was very different; guidelines were changing rapidly, the vaccine rollout was still in its early stages and aggressive public health control strategies (such as lockdowns and border closures) aimed at suppressing cases were in place. Fast forward to now and we’re learning to live with COVID-19. Today, we’re interested in finding out where your organisation is at now, compared to 12 months ago, and what you would like to see your organisation doing moving forward?
- Do you have any questions after reading the explanatory statement?
- Do you consent to participating in the interview?

**Professional role and impact of COVID-19 on work role/**

Can you tell me a little about your professional role and how COVID-19 has impacted on your work and your role in the past 12 months?

**Challenges and managing them**

Can you tell me about any challenges you’ve faced at work in the last 12 months, and how these compare to challenges you faced earlier in the pandemic?

- Financial? Feelings of isolation? Workflow?
- What helped? What didn’t help?
- What did your organisation do to address this? What else could they have done?

**COVID exposures**

Do you think the attitude of staff to COVID-19 risks and exposures has changed in the last 12 months?

- Can you tell me about these changes?
- If isolated/quarantined – experiences? Feelings on returning to work?
- What does your organisation do to support furloughed staff? What else could they be doing?

**Organisational responses**

Can you share some thoughts on how the leaders or senior personnel within your organisation have responded to the ongoing impacts of COVID over the past 12 months?

- What did you/your manager/leaders do that you/your staff appreciated?
- Was there anything you/they did that you think was not appreciated?

Can you share some thoughts about any changes to workload and workflow within your organisation in response to COVID-19 in the last 12 months?

- Staffing, resourcing, infrastructure, workflow etc
- How do you feel about these changes?

What support has your organisation offered to staff that you think has helped in the last 12 months? (e.g. to feel heard, safe, prepared, supported, cared for)

- Training? COVID-19 test access? Mental health and wellbeing?
- Do you think these supports will be sustained into the future?
- How did this help? Did you feel it was adequate? Are staff able to access it easily?

Can you talk about any opportunities you’ve had to suggest improvements in your organisation’s response and what came about from this?

Our latest round of survey findings indicates that 50% of HCWs who have completed the survey so far, have considered leaving their profession as a result of COVID-19, what are your thoughts on this?

Looking forward, what additional workplace supports would be helpful?

Is there anything else you’d like to say about your experiences of COVID-19 or your perspective on how your organisation has managed it?

**Closing**

- Thank you for your valuable contribution
- If you think of something you wish you’d said, please email [covicha@monash.edu](mailto:covicha@monash.edu). It will be added to your transcript
- Would you be willing to be contacted for another interview in the future?
- Collect demographic details ie. Role, age, gender & work experience (if not already stated)
